# Methyltransferase 3, N6-adenosine-methyltransferase complex catalytic subunit-induced long intergenic non-protein coding RNA 1833 N6-methyladenosine methylation promotes the non-small cell lung cancer progression via regulating heterogeneous nuclear ribonucleoprotein A2/B1 expression

**DOI:** 10.1080/21655979.2022.2061305

**Published:** 2022-04-20

**Authors:** Dongliang Li, Zejun Fu, Chaoqun Dong, Yongming Song

**Affiliations:** Department of Thoracic Surgery, Shanxi Provincial Cancer Hospital, hanxi Hospital Affiliated to Cancer Hospitalṣ Chinese Academy of Medical Sciences; Cancer Hospital Affiliated to Shanxi Medical University, Taiyuan, Shanxi 030000, China

**Keywords:** Non-small cell lung cancer, LINC01833, METTL3, m6A methylation, HNRNPA2B1, molecular mechanism

## Abstract

Long intergenic non-protein coding RNA 1833 (LINC01833) exhibits elevated expression in the non-small cell lung cancer (NSCLC) tissues, while its molecular mechanism in NSCLC progression remains elusive. Herein, the proliferation, migration, invasion as well as apoptosis of NSCLC cells were assessed. The potential N6-methyladenosine (m6A) modification site was predicted by the m6aVar tool. RNA pulldown and m6A-specific immunoprecipitation assays were used to detect the interaction between LINC01833 and methyltransferase 3, N6-adenosine-methyltransferase complex catalytic subunit (METTL3). RNA pull-down together with mass spectrometry were performed to assess the binding relationship between LINC01833 and heterogeneous nuclear ribonucleoprotein A2/B1 (HNRNPA2B1) in NSCLC. Tumor xenograft mice model was established, and the tumor size and weight were measured. The results demonstrated that LINC01833 expression was elevated in NSCLC samples. Overexpression of LINC01833 promoted proliferative, migratory, and invasive abilities and inhibited HCC827 cell apoptosis. LINC01833 knockdown inhibited tumor growth in mice. LINC01833 is further demonstrated to be modulated by METTL3, which is highly expressed in NSCLC samples. In addition, RNA pulldown and m6A-specific immunoprecipitation assays indicated that LINC01833 might form a complex with HNRNPA2B1. In conclusion, m6A transferase METTL3-induced LINC01833 m6A methylation promotes NSCLC progression through modulating HNRNPA2B1 expression. Our findings indicated that LINC01833 might be a therapeutic target for NSCLC.

## Graphical Abstract

**Figure:**
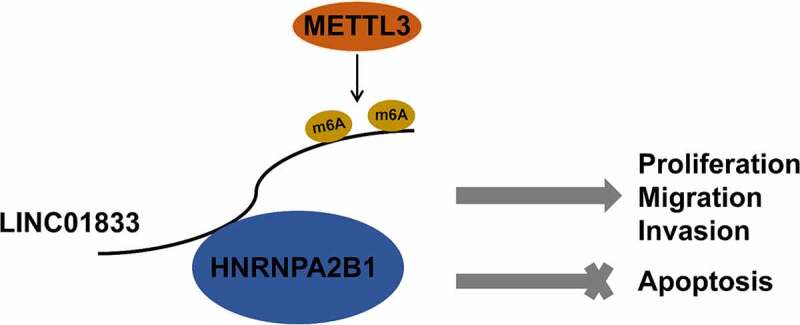

## 1. Introduction

Lung cancer is the most common malignancy worldwide, and its morbidity and mortality rank first in malignancies, of which non-small cell lung cancer (NSCLC) accounts for approximately 80% [1]. Since NSCLC has an insidious onset, the symptoms are not specific at the beginning. At present, the main treatment methods for lung cancer include surgical resection, chemotherapy, radiation therapy, targeted therapy, and immunotherapy that can greatly extend the survival time of patients [1,2]. However, because the pathogenesis of lung cancer is unknown and preventive and therapeutic methods are lacked, its morbidity and mortality are still high [3]. Therefore, exploring the mechanism of NSCLC and mining key regulatory factors are of great significance for the targeted therapy of NSCLC.

LncRNAs have over 200 nucleotides in length and lack the frame of open reading, with little or without protein coding ability [4,5]. It has been revealed that lncRNAs play vital roles in cell physiology and pathological activities and involve in occurrence and development of tumors [6,7]. Multiple studies have illustrated that lncRNAs exert vital roles in the NSCLC progression [8–10]. Liang et al. indicated that small nucleolar RNA host gene 10 is downregulated in NSCLC tissues and it predicts poor survival of patients with NSCLC [11]. Chen et al. found that competing endogenous lncRNA 2 for microRNA let-7b was highly expressed in NSCLC tissues and enhanced gefitinib-resistance in NSCLC by down-regulating miRNA-621 [12]. In addition, several lncRNAs were revealed as important biomarkers in NSCLC [13,14], such as long intergenic non-protein coding RNA 504 and long intergenic non-protein coding RNA 691 [15,16]. Recently, Yu et al successfully identified five survival-related lncRNAs using transcriptome profiling analysis of NSCLC from TCGA database [17]. Among them, the expression of long intergenic non-protein coding RNA 1833 (LINC01833) showed the most significant upregulation in tumors compared with normal group, suggesting that LINC01833 might act as an oncogene in NSCLC. However, the regulatory mechanism of LINC01833 remains unclear.

N6-methyladenosine (m6A) methylation widely exists in mammals and regulates gene expression after transcription without changing the base sequence [18]. m6A methylation affects tumor occurrence and development via regulation on proto-oncogene and tumor suppressor gene expression at the level of epigenetic modification through methyltransferase and demethyltransferase [19,20]. Several genes such as yes-associated protein and RNA component of mitochondrial RNA processing endoribonuclease were regulated by m6A methylation that participated in the development of lung cancer [21,22]. In addition, methyltransferase 3, n6-adenosine-methyltransferase complex catalytic subunit (METTL3) affects the tumor formation by regulating the m6A modification in lncRNAs [23–25].

Heterogeneous nuclear ribonucleoprotein A2/B1 (HNRNPA2B1) is a protein that ubiquitously participates in RNA‐binding and pre‐RNA processing, regulating cancer cell metabolism, proliferation, migration, invasion, and apoptosis [26–30]. HNRNPA2B1 shows high expression in lung cancers [31,32] and serves as a m6A regulator [33,34].

Based on bioinformatics analysis, there are m6A modification sites on LINC01833. We therefore hypothesized that METTL3 mediates m6A modification in LINC01833 to regulate NSCLC progression. Detailed functions of METTL3 and LINC01833 in NSCLC were further explored. Moreover, we provided an insight into the downstream molecular mechanism of LINC01833 in NSCLC.

## 2. Materials and methods

### 2.1 Bioinformatics analysis

The online tool m6aVar (http://m6avar.renlab.org/) was used to reveal the m6A modification sites on LINC01833. RNA binding proteins of LINC01833 were obtained from online tool Starbase (https://starbase.sysu.edu.cn/), which were listed based on the number of CLIP-seq experiments. HNRNPA2B1 was selected since it ranks the first.

### 2.2 NSCLC Specimen and cell lines

A cohort of 30 paired NSCLC specimens and adjacent tissues were obtained from Shanxi Provincial Cancer Hospital between December 2019 to December 2020. These specimens were immediately frozen and stored in liquid nitrogen. This study was approved by the Research Ethics Committee of Shanxi Provincial Cancer Hospital and informed consent was obtained from all patients. A copy of the ethical approval was provided in Supplementary material 1. NSCLC cell lines HCC827, NCI-H1299, A549 and NCI-H1650 and the normal lung epithelial cell BEAS-2B were obtained from COBIOER (Nanjing, China) and were cultured in Roswell Park Memorial Institute (RPMI) 1640 basic medium supplemented with 10% fetal bovine serum (FBS) in a humidified with 5% CO_2_ at 37°C.

### 2.3 Plasmid construction and cell transfection

The cDNA of LINC01833 or METTL3 was synthesized and cloned into the expression vector pcDNA3.1 (GenePharma, Shanghai, China). shRNA plasmids were designed by GenePharma to knock down LINC01833 or METTL3. The pCDNA3.1 vectors and shRNA plasmids were transfected into NSCLC cells HCC827 using RNAiMAX following the manufacturer’s manual after the cell confluence reached 70–90%. Cells were collected at 48 h post-transfection.

### 2.4 Reverse transcription-quantitative polymerase chain reaction (RT-qPCR)

Total RNA was extracted from tissue samples or cells using TRIZOL regent and the concentrations of total RNA were analyzed by Nanodrop. Reverse transcription of total RNA was performed using the PrimeScript RT Reagent Kit (Takara). The quantitative polymerase chain reaction (qPCR) was performed by ABI7500 using the SYBR Premix Ex Taq (Takara). Glyceraldehyde-3-phosphate dehydrogenase (GAPDH) was used as the internal control. The relative gene expression levels were calculated using the 2^−ΔΔCT^ method. The primers sequences are as follows: LINC01833-F, 5’-CATTACAGGCATCACCCAT-3’, LINC01833-R, 5’-GACAAACCCAGGTACCT-3’; HNRNPA2B1-F, 5’-CCAGGGACATTTACTCAGACCAAT-3’, HNRNPA2B1-R, 5’-GGACACCTACCTTTATCATTCCC-3’; U6-F, 5’-TACTTACCCAGGACCAGG-3’, U6-R, 5’-CCATTAGACCTGGACTG-3’; GAPDH-F, 5’-GGACCTGCATTACTCCGATCT-3’, GAPDH-R, 5’-CGGGCCATATTACATACT-3’.

### 2.5 Western blot

Proteins were isolated by RIPA buffer (Beyotime) and determined using a BCA protein kit (Beyotime). Thirty µg protein was loaded into a 10% sodium dodecyl sulfate polyacrylamide gel and then electrophoresed, transferred onto polyvinylidene difluoride membranes. The membranes were blocked with 5% fat-free milk for 2 h at room temperature and incubated with primary antibodies against METTL3 and HNRNPA2B1 overnight at 4°C. Subsequently, the membranes were incubated with a horseradish peroxidase-conjugated immunoglobulin G secondary antibody for 2 h at room temperature. Enhanced chemiluminescence reagent was applied to the membranes for visualization. Protein bands were analyzed by Quantity One software version 4.62 (Bio-Rad). Original Western blot images were provided in Supplementary material 2.

### 2.6 Cell counting kit-8 (CCK-8) assay

The cells with different transfections were incubated at 37°C in a 5% CO_2_ incubator for 24 h and then were harvested, resuspended in a culture medium, and seeded into 96-well plates at a density of 2 × 10^3^ cells/well. Cell proliferation was assessed by incubating cells with 10 µl of CCK-8 solution for 0, 24, 48, and 72 h at 37°C for 2 h. The optical density (OD) was measured at 450 nm using a Tecan microplate reader.

### 2.7 Flow cytometric assays

For cell apoptosis analysis, cells were harvested at 48 h after transfection. Propidium iodide (PI) and fluorescein isothiocyanate (FITC)-Annexin V were used to stain the cells in the dark. Cell apoptosis was measured by a flow cytometer (FACScan; BD Biosciences). Original flow cytometry data were provided in Supplementary material 3.

### 2.8 Cell migration and invasion assays

Transfected cells were collected and suspended to a density of 1 × 10^5^ cells/ml. For cell migration assay, upper chamber was equipped with 8-µm porous membranes (BD Biosciences) and added with 100 µl of the suspension. Six hundred µl of complete culture medium was added to each lower chamber to induce migration. The non-migrated cells were removed with a cotton swab after incubation for 24 h at 37°C with 5% CO_2_ whereas the migrated cells were fixed with 4% (v/v) paraformaldehyde for 20 min and stained for 20 min with 0.1% crystal violet. The number of migrated cells was taken by an inverted microscope. For the cell invasion assay, chambers were precoated with Matrigel (BD Biosciences).

### 2.9 RNA immunoprecipitation (RIP) and methylated RNA immunoprecipitation (MeRIP)

RIP assays were conducted using an EZ Magna RIP kit (Millipore) with the Protein A/G Agarose Beads (Santa Cruz) according to the manufacturer’s instructions. The cells were lysed, and the lysates were incubated with magnetic beads conjugated with the HNRNPA2B1-specific antibody or control IgG antibody for 36 h at 4°C. The beads were incubated with proteinase K after washing with wash buffer, the purified RNA was eluted and analyzed for the presence of LINC01833 4 by qRT-PCR.

### 2.10. RNA m6A quantification

The m6A content in the total RNAs was measured using an m6A RNA methylation quantification kit (ab185912; Abcam). Two hundred ng RNAs were added into the assay wells. By detecting the absorbance at wavelength of 450 nm, the m6A content was quantified following the manufacturer’s protocol.

### 2.11 RNA pull-down assays

LINC01833 or its antisense RNA was transcribed *in vitro* using T7 RNA polymerase and purified using RNA PURE Kit. The transcribed LINC01833 RNA was biotinylated with a Biotin RNA Labeling Mix. Biotinylated RNAs were incubated with streptavidin-conjugated magnetic beads at room temperature. Cell was lyzed, the beads were then washed, and the eluted proteins were examined by mass spectrometry and Western blot analysis.

### 2.12 In vivo tumor bearing mice model experiments

Male BALB/c nude mice (4 weeks old) were purchased from Beijing Vital River Company and fed in a specified pathogen-free environment. For the tumorigenicity studies, 3 × 10^6^ HCC827 cells stably expressing sh-LINC01833 or sh-NC were injected subcutaneously into the ventral side of male BALB/c nude mice in the according groups with five mice in each group. Five mice were sampled each time. Tumor size and weight were examined at 28 days after injection. The volume was calculated as follows: V (mm^3^) = 0.5 × D × d^2^ (V is volume; D is longest diameter, and d is diameter perpendicular to the longest diameter). The tumors were excised, weighed, and collected for IHC staining. The test results were repeated three times. All protocols were approved by the Committee on the Ethics of Animal Experiments of the Shanxi Provincial Cancer Hospital and performed in accordance with the Guide for the Care and Use of Laboratory Animals of the National Institutes of Health.

### 2.13 Immunohistochemistry (IHC)

The tumor tissues were formalin-fixed, paraffin-embedded and cut into 3-μm sections. Next, the sections were deparaffinated with xylene and rehydration, and 10 mM citrate buffer (pH 6.0) was used for antigen retrieval for 20 min. Endogenous peroxidase activity was blocked by 0.3% hydrogen peroxide. Next, samples were incubated with primary antibodies against METTL3 and HNRNPA2B1 overnight at 4°C and the biotinylated goat anti-mouse or goat anti-rabbit IgG secondary antibody (Dako) for 30 min. Next, the sections were incubated with streptavidin peroxidase for 30 min. The slides were stained with diaminobenzidine (DAB) and counterstained with hematoxylin, dehydrated by a graded ethanol series, and photographed.

### 2.14 Statistical analysis

The statistical analysis was performed using SPSS 21.0 software and data were presented as a mean ± standard deviation (SD). There are 3 technical repeats for all assays. An equivalent non-parametric test, the Kruskal–Wallis Test, was performed to test the assumption of normality. Homogeneity of variance was tested using Bartlett’s test. Comparisons between two or more groups were detected using Student’s t-test or one-way ANOVA followed by Tukey’s post-hoc test. A P value of less than 0.05 was considered to be statistically significant.

## 3. Results

### 3.1 LINC01833 shows upregulation in NSCLC and promotes NSCLC progression

**Figure 1.**
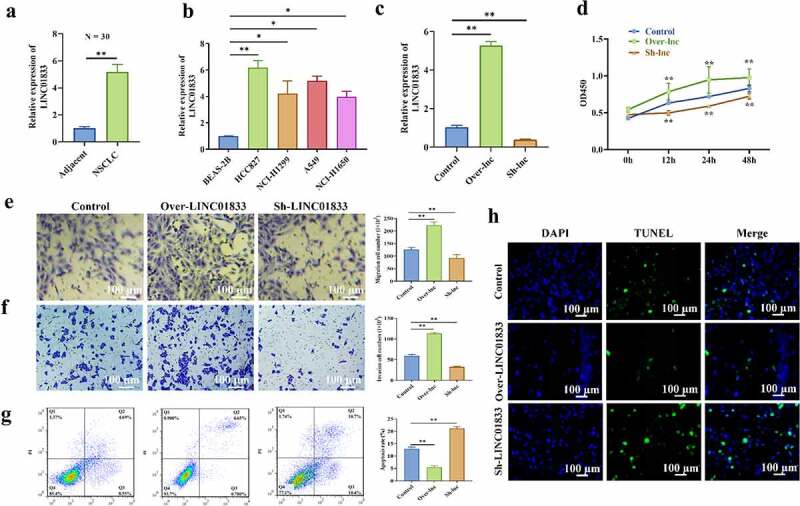
**LINC01833 shows upregulation in NSCLC tissue samples or cells and promotes NSCLC progression**. (a-b) qRT-PCR was performed to detect LINC01833 expression level in the NSCLC tissue samples (n = 30) and NSCLC cells; (c) LINC01833 was overexpressed or suppressed in the HCC827 cells by transfection with over- or sh-LINC01833 plasmid; (d) CCK-8 was used to detect HCC827 cell proliferation under the transfection of over-LINC01833 and sh-LINC01833; (e-f) Transwell assays were used to detect cell migration and invasion in over-LINC01833 group and sh-LINC01833 group; (g-h) Flow cytometry and TUNEL were performed to detect cell apoptosis. Data were expressed as a mean ± SD. There are 3 technical repeats. *, P < 0.05, **, P < 0.01.

The expression profile of LINC01833 and its functions in NSCLC were investigated. The results showed that LINC01833 expression was elevated in 30 NSCLC tissues (Figure 1a). Additionally, LINC01833 presented upregulation in NSCLC cell lines compared with the control cells. LINC01833 showed the most upregulation in the HCC827 cells (Figure 1b). Therefore, HCC827 cells was used for further studies. Subsequently, LINC01833 expression was overexpressed or suppressed in the HCC827 cells by transfection with over- or sh-LINC01833 plasmid. LINC01833 was successfully overexpressed or suppressed after transfection (Figure 1c). HCC827 cell proliferation was obviously increased in over-LINC01833 group and reduced in sh-LINC01833 group (Figure 1d). Transwell assay results revealed that migratory and invasive abilities were promoted in over-LINC01833 group and inhibited in sh-LINC01833 group (Figure 1e, f). On contrast, the apoptosis rates were significantly decreased in over-LINC01833 group while increased in sh-LINC01833 group compared with NC group (Figure 1g). This result was also confirmed by TUNEL assay (Figure 1h). These results suggested that LINC01833 promoted the NSCLC progression.

### 3.2 LINC01833 is modulated by m6A RNA methylation

**Figure 2.**
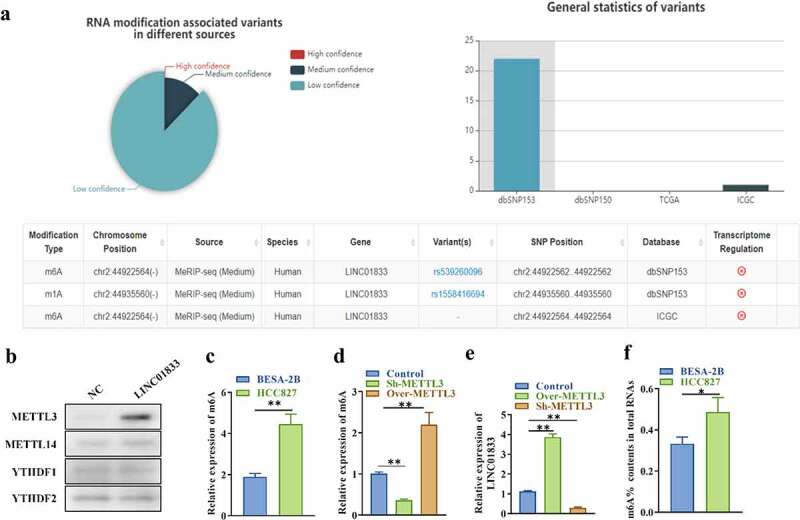
**LINC01833 is modulated by m6A RNA methylation**. (a) The potential m6A modification sited was predicted using bioinformatics (m6aVar, http://m6avar.renlab.org/); (b) RNA pull-down was used to detect relationship of LINC01833 and METTL3, METTL14, FTO and YTHDF1/2; (c-d) m6A-specific immunoprecipitation assay was used to detect m6A level of LINC01833 in HCC827 cells with overexpression or knockdown of METTL3; (e) The expression of LINC01833 under the overexpression or knockdown of METTL3 was detected by qRT-PCR. F. m6A contents (%) in total RNAs of HCC827 cells and BEAS-2B cells. Data were expressed as a mean ± SD. There are 3 technical repeats. *, P < 0.05, **, P < 0.01.

In order to know whether LINC01833 is modulated by m6A RNA methylation, the potential m6A modification site was predicted using m6aVar online tool. There was three potential m6A modification sites on LINC01833 sequence (Figure 2a), suggesting that m6A modification might exert important effects on regulating LINC01833 during NSCLC progression. METTL3, methyltransferase 14, N6-adenosine-methyltransferase complex catalytic subunit (METTL14) and YTH N6-methyladenosine RNA binding protein 1/2 (YTHDF1/2) are m6A modifiers in human NSCLC and play oncogenic roles in this cancer [35]. Results of RNA pulldown assays indicated that LINC01833 could interact with m6A modifier METTL3 while could not interact with METTL14 and YTHDF1/2 (Figure 2b). Results of m6A-specific immunoprecipitation assays indicated that the m6A levels of LINC01833 were increased in HCC827 cells than that in BEAS-2B cells (Figure 2c). m6A levels were reduced by sh-METTL3 and increased by over-METTL3 in HCC827 cells (Figure 2d). LINC01833 showed significantly higher level under METTL3 overexpression and lower level under METTL3 depletion in HCC827 cells (Figure 2e). Additionally, the m6A content (%) in total RNAs was higher in HCC827 than BEAS-2B (figure 2f). These findings indicated that LINC01833 is modulated by METTL3-mediated m6A RNA methylation.

### 3.3 METTL3 shows elevation in NSCLC and promotes NSCLC progression

**Figure 3.**
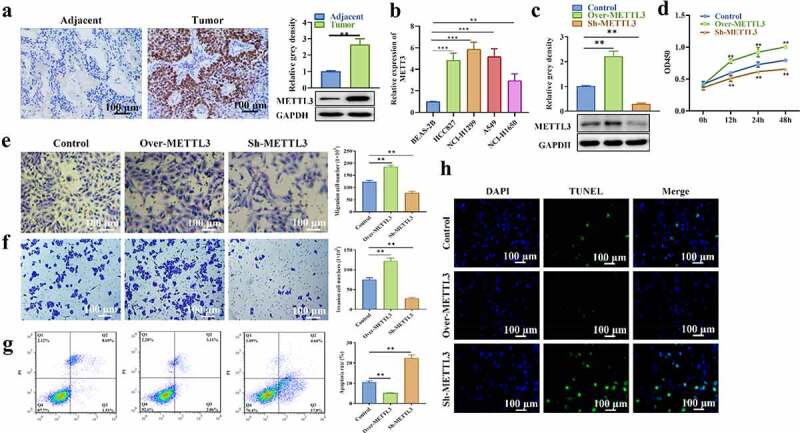
**METTL3 shows elevation in NSCLC tissue samples and cells and promotes NSCLC progression**. (a-b) IHC and Western blot were performed to measure METTL3 level in NSCLC tissues; (c) The expression of LINC01833 was successfully overexpressed or suppressed, which is detected by qRT-PCR; (d) CCK-8 was used to detect HCC827 cell proliferation affected by METTL3; (e-f) Transwell assay was used to detect HCC827 cell migration and invasion that is affected by METTL3, respectively; (g-h) Flow cytometry and TUNEL were used to detect cell apoptosis that is affected by METTL3. Data were expressed as a mean ± SD. There are 3 technical repeats. **, P < 0.01.

The expression profile of LINC01833 and its functions in NSCLC were subsequently explored. METTL3 level in NSCLC tissues was detected with IHC as well as Western blot. METTL3 presented upregulation in NSCLC tissues and cell lines (Figures 3A and 3b). METTL3 was successfully overexpressed or suppressed after transfection with over-METTL3 or sh-METTL3 (Figure 3c). CCK-8 assay results illustrated that HCC827 cell proliferation was obviously increased in over-METTL3 group while reduced in sh-METTL3 group (Figure 3d). Transwell assay results showed that METTL3 elevation promoted HCC827 cell migratory and invasive abilities while METTL3 depletion inhibited migration and invasion compared with NC group (Figures 3E and 3f). On contrast, the apoptosis rates were significantly decreased in over-METTL3 group while increased in sh-METTL3 group compared with NC group (Figure 3g). This result was also confirmed by TUNEL assay (Figure 3h). These results suggested that METTL3 promoted the NSCLC progression.

### 3.4 LINC01833 binds with HNRNPA2B1 in NSCLC cells

**Figure 4.**
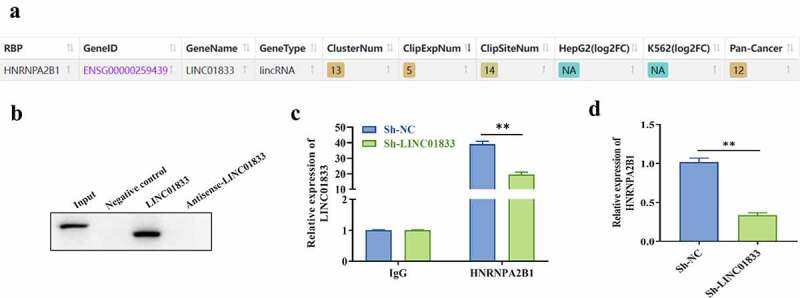
**LINC01833 binds with HNRNPA2B1 in NSCLC cells**. (a) The binding relationship between LINC01833 and the RBPs was examined by Starbase; (b) RNA pull down was used to verify binding of LINC01833 and HNRNPA2B1; (c) RIP assay was used to detect the expression of LINC01833 that is enriched in anti-HNRNPA2B1-formed precipitates; (d) qRT-PCR was used to detect the expression of HNRNPA2B1 affected by LINC01833. Data were expressed as a mean ± SD. There are 3 technical repeats. **, P < 0.01.

To clarify the regulatory mechanism of LINC01833, the RNA binding proteins of LINC01833 were examined by Starbase. The results indicated that HNRNPA2B1 is a potential RBP of LINC01833 (Figure 4a). Subsequently, the direct binding function between LINC01833 and HNRNPA2B1 protein was confirmed by RNA pull-down assays. HNRNPA2B1 was pulled down by biotinylated LINC01833 rather than negative control or antisense LINC01833 (Figure 4b). RIP assay results demonstrated that LINC01833 presented elevation in HNRNPA2B1 group. After knockdown of LINC01833, the binding of LINC01833 and HNRNPA2B1 protein was weakened (Figure 4c). In addition, we found that the expression of HNRNPA2B1 was reduced when knocking down LINC01833 (Figure 4d).

### 3.5 Inhibition of LINC01833 alleviated tumorigenesis

**Figure 5.**
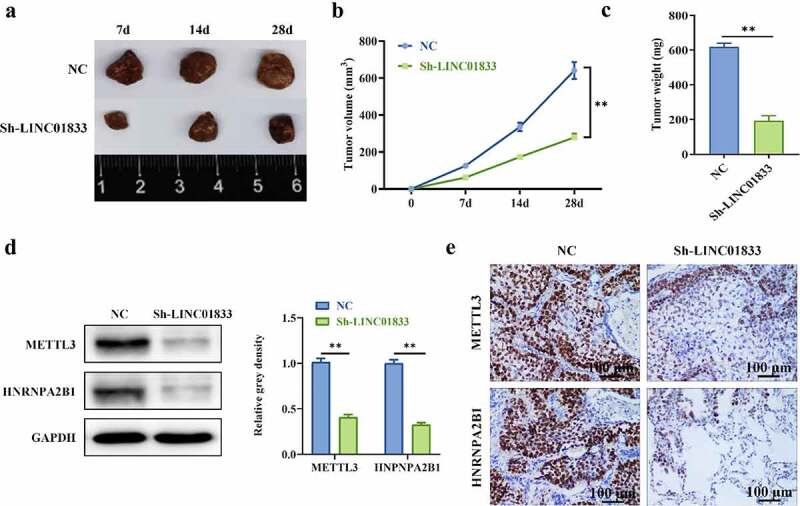
**Inhibition of LINC01833 alleviated tumorigenesis**. (a) LINC01833 expression was reduced in sh-LINC01833 group compared with NC group; (b-c) The tumor size and weight were reduced under LINC01833 knockdown; (d-e) The expression levels of METTL3 and HNRNPA2B1 were detected by Western blot and IHC in tumor tissues. Data were presented as a mean ± SD. Each group has 5 mice. There are 3 technical repeats. **, P < 0.01.

Role of LINC01833 was further investigated by knocking down LINC01833 in tumor xenograft mice model. The expression of LINC01833 was reduced in sh-LINC01833 group (Figure 5a). The tumor size and weight were reduced under LINC01833 knockdown (Figures 5B and 5c). Additionally, Western blot and IHC were conducted to measure METTL3 and HNRNPA2B1 protein levels. METTL3 and HNRNPA2B1 expression was reduced in tumor xenografts with LINC01833 suppression (Figures 5D and 5e). These findings suggested that knockdown of LINC01833 suppresses NSCLC progression *in vivo*.

## 4. Discussion

Multiple reports have indicated that lncRNAs exert crucial impacts on various kinds of cancer progression by regulating different cellular processes, including cycle, migration, apoptosis, and physiological changes [36]. In the previous studies, Yu et al. identified five lncRNAs which showed important prognostic values in NSCLC. LINC01833 showed the most significant upregulation in the NSCLC tissues compared with control group [17]. The present study shows that overexpression of LINC01833 promoted cell proliferative, invasive, and migratory capabilities, inhibited cell apoptosis, and promoted tumorigenesis, suggesting that LINC01833 can promote NSCLC progression.

m6A modification at the post-transcriptional level in the eukaryotic genome exerts an important role in the pathophysiological process of multiple cancers [18,19]. Li et al. indicated that patients with FTO and YTHDC2 deficiency have unfavorable disease-free survival and overall survival, suggesting the prognostic value of these m6A RNA methylation regulators in NSCLC [37]. Yin et al. showed that m6A methylation controls RMRP stability to render NSCLC progression [22]. Recently, Lei et al. showed that ABHD11-AS1 showed upregulation in NSCLC tissue samples and cells. METTL3 stabilizes ABHD11-AS1 transcript to upregulate ABHD11-AS1 by installing the m6A modification [24]. In the present study, we found that LINC01833 is modulated by METTL3-mediated m6A RNA methylation, suggesting that the METTL3-medicated m6A RNA methylation plays crucial roles in the regulation of LINC01833 on NSCLC progression.

HNRNPA2B1 is an important RNA binding protein affecting the RNA splicing and transport and stability of its downstream target genes. HNRNPA2B1 exerts crucial impacts on lung cancers. Dai et al. [32] and Dowling et al. [38] demonstrated that abnormal levels of HNRNPA2B1 could act as an important diagnose indicator for lung cancer patients. Yu et al. demonstrated that CACNA1G-AS1 promoted NSCLC cell migratory and invasive abilities through regulating HNRNPA2B1 [39]. Recently, Jin et al. indicated that HNRNPA2B1 promoted NSCLC malignancy through facilitating cell growth and metastasis, suggesting that HNRNPA2B1 is a promising targeted biomarker for therapy of NSCLC [40]. Herein, LINC01833 could bind to HNRNPA2B1 in NSCLC cells. In consistent with previous reports, HNRNPA2B1 overexpression promoted NSCLC cell proliferative, invasive, and migratory capabilities and inhibited apoptosis. In addition, HNRNPA2B1 is recognized as a modulator of m6A-dependent RNA processing events in nucleus. HNRNPA2B1 directly interacts with a set of nuclear transcripts and elicits similar alternative splicing function as the m6A writer METTL3 [41]. Therefore, we concluded that METTL3-induced LINC01833 promoted the NSCLC progression by interacting with HNRNPA2B.

Interestingly, it was found that the upstream modulator of LINC01833, METTL3, is suppressed by LINC01833 knockdown in *in vivo* studies. HNRNPA2B1 is an RNA binding protein and may bind with mRNAs to stabilize or destabilize their expression. We used the starbase tool to identify the downstream targets of HNRNPA2B1 and found 11,130 mRNAs. Proteins of 100 mRNAs among these 11,130 mRNAs were predicted as transcription factors for METTL3 (Supplementary material 4). LINC01833 suppression-induced reduction of METTL3 may be associated with the 100 genes that were regulated by HNRNPA2B1 (positively regulated by LINC01833) and spontaneously modulated METTL3.

## Conclusion

m6A transferase METTL3-triggered LINC01833 m6A methylation facilitates NSCLC progression through modulating HNRNPA2B1, which provides a novel direction for the better understanding of the pathogenesis of NSCLC.

## Supplementary Material

Supplemental Material

## References

[1] Oberndorfer F, Mullauer L. Molecular pathology of lung cancer: current status and perspectives. Curr Opin Oncol. 2018;30:69–76. 29251665 10.1097/CCO.0000000000000429

[2] Rodriguez-Canales J, Parra-Cuentas E, Wistuba II. Diagnosis and molecular classification of lung cancer. Cancer Treat Res. 2016;170:25–46. 27535388 10.1007/978-3-319-40389-2_2

[3] Bade BC, Dela Cruz CS. Lung cancer 2020: epidemiology, etiology, and prevention. Clin Chest Med. 2020;41:1–24. 32008623 10.1016/j.ccm.2019.10.001

[4] Groen JN, Capraro D, Morris KV. The emerging role of pseudogene expressed non-coding RNAs in cellular functions. Int J Biochem Cell Biol. 2014;54:350–355. 24842102 10.1016/j.biocel.2014.05.008 PMC4160391

[5] Peng WX, Koirala P, Mo YY. LncRNA-mediated regulation of cell signaling in cancer. Oncogene. 2017;36:5661–5667. 28604750 10.1038/onc.2017.184 PMC6450570

[6] Chan JJ, Tay Y. Noncoding RNA:RNA regulatory networks in cancer. Int J Mol Sci. 2018;19:1310. 10.3390/ijms19051310 PMC5983611 29702599

[7] Jathar S, Kumar V, Srivastava J, et al. Technological Developments in lncRNA Biology. Adv Exp Med Biol. 2017;1008:283–323. 28815544 10.1007/978-981-10-5203-3_10

[8] Tan D, Li G, Zhang P, et al. LncRNA SNHG12 in extracellular vesicles derived from carcinoma-associated fibroblasts promotes cisplatin resistance in non-small cell lung cancer cells. Bioengineered. 2022;13:1838–1857. 35014944 10.1080/21655979.2021.2018099 PMC8805932

[9] Zheng Y, Guo Z, Li Y. Long non-coding RNA prostate cancer-associated transcript 6 inhibited gefitinib sensitivity of non-small cell lung cancer by serving as a competing endogenous RNA of miR-326 to up-regulate interferon-alpha receptor 2. Bioengineered. 2022;13:3785–3796. 35081872 10.1080/21655979.2022.2031416 PMC8974150

[10] Tang X, Wu Y, Yang J, et al. Regulating COX10-AS1/miR-142-5p/PAICS axis inhibits the proliferation of non-small cell lung cancer. Bioengineered. 2021;12:4643–4653. 34323174 10.1080/21655979.2021.1957072 PMC8806450

[11] Liang M, Wang L, Cao C, et al. LncRNA SNHG10 is downregulated in non-small cell lung cancer and predicts poor survival. BMC Pulm Med. 2020;20:273. 33081752 10.1186/s12890-020-01281-w PMC7574240

[12] Chen ZY, Liu HY, Jiang N, et al. LncRNA HOST2 enhances gefitinib-resistance in non-small cell lung cancer by down-regulating miRNA-621. Eur Rev Med Pharmacol Sci. 2019;23:9939–9946. 31799663 10.26355/eurrev_201911_19560

[13] Chen J, Wang R, Zhang K, et al. Long non-coding RNAs in non-small cell lung cancer as biomarkers and therapeutic targets. J Cell Mol Med. 18:2425-36 2014;18(12):2014 10 09 .DOI:10.1111/jcmm.12431. PMC4302648 25297942

[14] Zhang Y, Liang D, Jin J, et al. Progress of long non-coding rna in non-small cell lung cancer. Zhongguo Fei Ai Za Zhi. 2018;21:43–49. 29357972 10.3779/j.issn.1009-3419.2018.01.06 PMC5972355

[15] Ma HP, Wang LX, Li W, et al. Upregulation of LINC00504 is associated with aggressive progression and poor prognosis in non-small cell lung cancer. Eur Rev Med Pharmacol Sci. 2020;24:699–703. 32016971 10.26355/eurrev_202001_20047

[16] Xie Y, Hu X. Increased levels of long noncoding RNA LINC00691 correlate with poor prognosis in non-small-cell lung cancer patients. J Clin Lab Anal. 2020;34:e23357. 32420681 10.1002/jcla.23357 PMC7439350

[17] Yu Y, Ren K. Five long non-coding RNAs establish a prognostic nomogram and construct a competing endogenous RNA network in the progression of non-small cell lung cancer. BMC Cancer. 2021;21:457. 33892664 10.1186/s12885-021-08207-7 PMC8067646

[18] Zhang CY, Zhang J, Ma YF, et al. Prognostic value of combined analysis of CTLA-4 and PLR in esophageal squamous cell carcinoma (escc) patients. Dis Markers. 2019;2019:1601072. 31485274 10.1155/2019/1601072 PMC6710793

[19] Wang T, Kong S, Tao M, et al. The potential role of RNA N6-methyladenosine in Cancer progression. Mol Cancer. 2020;19:88. 32398132 10.1186/s12943-020-01204-7 PMC7216508

[20] Zhang C, Fu J, Zhou Y. A review in research progress concerning m6a methylation and immunoregulation. Front Immunol. 2019;10:922. 31080453 10.3389/fimmu.2019.00922 PMC6497756

[21] Jin D, Guo J, Wu Y, et al. m(6)A mRNA methylation initiated by METTL3 directly promotes YAP translation and increases YAP activity by regulating the MALAT1-miR-1914-3p-YAP axis to induce NSCLC drug resistance and metastasis. J Hematol Oncol. 2021;14:32. 33618740 10.1186/s13045-021-01048-8 PMC7901070

[22] Yin H, Chen L, Piao S, et al. M6A RNA methylation-mediated RMRP stability renders proliferation and progression of non-small cell lung cancer through regulating TGFBR1/SMAD2/SMAD3 pathway. Cell Death Differ. 2021. DOI:10.1038/s41418-021-00888-8. PMC9984538 34628486

[23] Liu HT, Zou YX, Zhu WJ, et al. lncRNA THAP7-AS1, transcriptionally activated by SP1 and post-transcriptionally stabilized by METTL3-mediated m6A modification, exerts oncogenic properties by improving CUL4B entry into the nucleus. Cell Death Differ. 2022;29:627–641. 34608273 10.1038/s41418-021-00879-9 PMC8901790

[24] Xue L, Li J, Lin Y, et al. m(6) A transferase METTL3-induced lncRNA ABHD11-AS1 promotes the Warburg effect of non-small-cell lung cancer. J Cell Physiol. 2021;236:2649–2658. 32892348 10.1002/jcp.30023

[25] Jin D, Guo J, Wu Y, et al. m(6)A mRNA methylation initiated by METTL3 directly promotes YAP translation and increases YAP activity by regulating the MALAT1-miR-1914-3p-YAP axis to induce NSCLC drug resistance and metastasis. J Hematol Oncol. 2019;12:135. 31818312 10.1186/s13045-019-0830-6 PMC6902496

[26] Clower CV, Chatterjee D, Wang Z, et al. The alternative splicing repressors hnRNP A1/A2 and PTB influence pyruvate kinase isoform expression and cell metabolism. Proc Natl Acad Sci U S A. 2010;107:1894–1899. 20133837 10.1073/pnas.0914845107 PMC2838216

[27] He Y, Brown MA, Rothnagel JA, et al. Roles of heterogeneous nuclear ribonucleoproteins A and B in cell proliferation. J Cell Sci. 2005;118:3173–3183. 16014382 10.1242/jcs.02448

[28] He Y, Rothnagel JA, Epis MR, et al. Downstream targets of heterogeneous nuclear ribonucleoprotein A2 mediate cell proliferation. Mol Carcinog. 2009;48:167–179. 18680105 10.1002/mc.20467

[29] Moran-Jones K, Grindlay J, Jones M, et al. hnRNP A2 regulates alternative mRNA splicing of TP53INP2 to control invasive cell migration. Cancer Res. 2009;69:9219–9227. 19934309 10.1158/0008-5472.CAN-09-1852 PMC6485436

[30] Patry C, Bouchard L, Labrecque P, et al. Small interfering RNA-mediated reduction in heterogeneous nuclear ribonucleoparticule A1/A2 proteins induces apoptosis in human cancer cells but not in normal mortal cell lines. Cancer Res. 2003;63:7679–7688. 14633690

[31] Zhang Y, Huang W, Yuan Y, et al. Long non-coding RNA H19 promotes colorectal cancer metastasis via binding to hnRNPA2B1. J Exp Clin Cancer Res. 2020;39:141. 32698890 10.1186/s13046-020-01619-6 PMC7412843

[32] Dai L, Li J, Tsay JJ, et al. Identification of autoantibodies to ECH1 and HNRNPA2B1 as potential biomarkers in the early detection of lung cancer. Oncoimmunology. 2017;6:e1310359. 28638733 10.1080/2162402X.2017.1310359 PMC5467997

[33] Jin L, Chen C, Yao J, et al. The RNA N(6) -methyladenosine modulator HNRNPA2B1 is involved in the development of non-small cell lung cancer. Clin Exp Pharmacol Physiol. 2022;49:329–340. 34717005 10.1111/1440-1681.13608

[34] Zhao Z, Wan J, Guo M, et al. Expression and prognostic significance of m6A-related genes in TP53-mutant non-small-cell lung cancer. J Clin Lab Anal. 2022;36:e24118. 34812534 10.1002/jcla.24118 PMC8761469

[35] Huang H, Weng H, Chen J. m(6)a modification in coding and non-coding RNAS: roles and therapeutic implications in cancer. Cancer Cell. 2020;37:270–288. 32183948 10.1016/j.ccell.2020.02.004 PMC7141420

[36] Braicu C, Zimta AA, Harangus A, et al. The Function of Non-Coding RNAs in Lung Cancer tumorigenesis. Cancers (Basel). 2019;11 4:605. doi:10.3390/cancers11050605. Published 2019 Apr 30 . PMC6563001 31052265

[37] Li N, Chen X, Liu Y, et al. Gene characteristics and prognostic values of m(6)A RNA methylation regulators in nonsmall cell lung cancer. J Healthc Eng. 2021;2021:2257066. 34367534 10.1155/2021/2257066 PMC8346307

[38] Dowling P, Pollard D, Larkin A, et al. Abnormal levels of heterogeneous nuclear ribonucleoprotein A2B1 (hnRNPA2B1) in tumour tissue and blood samples from patients diagnosed with lung cancer. Mol Biosyst. 2015;11:743–752. 25483567 10.1039/c4mb00384e

[39] Yu PF, Kang AR, Jing LJ, et al. Long non-coding RNA CACNA1G-AS1 promotes cell migration, invasion and epithelial-mesenchymal transition by HNRNPA2B1 in non-small cell lung cancer. Eur Rev Med Pharmacol Sci. 2018;22:993–1002. 29509247 10.26355/eurrev_201802_14381

[40] Jin L, Chen C, Yao J, Yu Z, Bu L, et al. The RNA N(6) -methyladenosine modulator HNRNPA2B1 is involved in the development of non-small cell lung cancer. Clin Exp Pharmacol Physiol. 2021;49(3):329–340. doi:10.1111/1440-1681.13608 . 34717005

[41] Alarcon CR, Goodarzi H, Lee H, et al. HNRNPA2B1 is a mediator of m(6)A-dependent nuclear rna processing events. Cell. 2015;162:1299–1308. 26321680 10.1016/j.cell.2015.08.011 PMC4673968

