# Supplementary figures and images for: Methyltransferase 3, N6-adenosine-methyltransferase complex catalytic subunit-induced long intergenic non-protein coding RNA 1833 N6-methyladenosine methylation promotes the non-small cell lung cancer progression via regulating heterogeneous nuclear ribonucleoprotein A2/B1 expression
Source: Bioengineered. 2022 Apr 20;13(4):10493–503. doi: 10.1080/21655979.2022.2061305 (PMC9161889; doi:10.1080/21655979.2022.2061305)

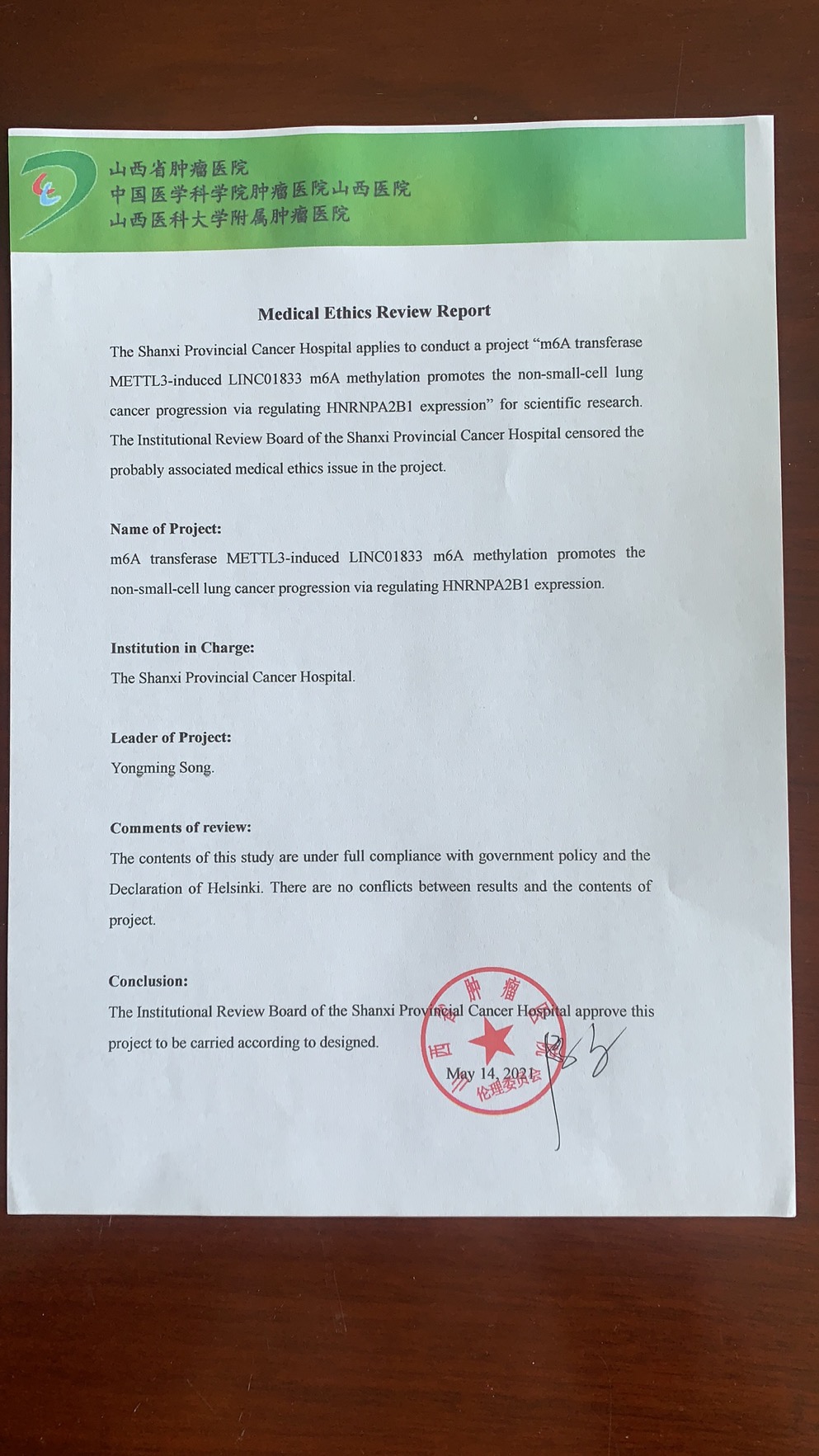

Supplement: Supplemental Material [file KBIE_A_2061305_SM8250.zip › supplementary/Supplementary material 1.jpg]
